# Evolution and Vaccine Strain Match of *HA* and *NA* Genes of Influenza A/H3N2 Subtype in Riyadh, Saudi Arabia, 2020–2023

**DOI:** 10.3390/vaccines13121184

**Published:** 2025-11-22

**Authors:** Noorah A. Alkubaisi, Ibrahim M. Aziz, Mohamed A. Farrag, Reem M. Aljowaie, Asma N. Alsaleh, Fatimah N. Alanazi, Fahad N. Almajhdi

**Affiliations:** Department of Botany and Microbiology, College of Science, King Saud University, P.O. Box 2455, Riyadh 11451, Saudi Arabia; nalkubaisi@ksu.edu.sa (N.A.A.); mfarrag@ksu.edu.sa (M.A.F.); raljowaie@ksu.edu.sa (R.M.A.); asmalsaleh@ksu.edu.sa (A.N.A.); 445205516@student.ksu.edu.sa (F.N.A.)

**Keywords:** influenza, vaccine effectiveness, infection, phylogenetic analysis, molecular epidemiology

## Abstract

**Background/Objectives**: Although several studies have shed light on the epidemiology of the influenza A/H3N2 subtype in Saudi Arabia, the knowledge regarding the molecular epidemiology and genetic diversity of the A/H3N2 subtype in the Riyadh region is still significantly restricted. Thus, the current research intends to investigate the molecular epidemiology and circulation patterns of the influenza A/H3N2 subtype in Riyadh, Saudi Arabia, over the past 9 years. **Methods**: A total of 380 nasopharyngeal aspirate samples (NPAs) (winter seasons 2020–2023) were screened for the presence of A/H3N2 subtype. **Results**: Sixty-five samples (17.11%) were found to be positive for the influenza A virus (IAV). A/H3N2 subtype 35 (9.21%) slightly predominated over A/H1N1 pdm09 30 (7.89%), the incidence rate was high in males (16.47%), and the most affected group was the 0–4 age group (14, 14.75%). The phylogenetic analysis revealed that the majority of Riyadh A/H3N2 samples were categorized into the sub-clades 3c.2a1b.1a and 3c.2a1b.1b, which did not exhibit any exclusive clustering with the vaccine strains. Out of the 20 amino acid substitutions detected in the HA1 domain of A/H3N2 strains, 9 were not found in any of the vaccine strains. The HA protein from the Riyadh samples has 8–11 N-glycosylation sites, some of which have been recorded in vaccine strains, yet are lacking in all strains analyzed in this study. **Conclusions**: As a result, the flu vaccines administered in Saudi Arabia might need to be reevaluated to incorporate additional vaccine strains that are more pertinent to those currently circulating in the recent epidemic seasons in Saudi Arabia.

## 1. Introduction

Influenza is a major respiratory disease that causes significant annual morbidity and mortality in animals and humans. The World Health Organization (WHO) approximates that around 5–10% of adults and 20–30% of children are infected with influenza each year, leading to 3–5 million cases of severe illness and roughly 1 million fatalities globally [1]. According to the International Committee on Taxonomy of Viruses (ICTV), Influenza viruses are members of the *Orthomyxoviridae* family, which are characterized by having a negative-sense, segmented RNA genome [2]. Humans are generally infected by three types of influenza viruses: influenza A virus (IAV), influenza B virus (IBV), and influenza C virus (ICV) [3], with type A posing the most significant risk to human health [4].

The A(H3N2) subtype, which emerged in 1968, was associated with increased rates of influenza morbidity and mortality worldwide until 1972. Since then, this subtype has continued to circulate as a seasonal IAV, leading to more severe annual epidemics compared to those caused by influenza A(H1N1) and IBV [5]. Common antigenic drift variants appear in H3 of A/H3N2 viruses [6].

Seasonal influenza A/H3N2 viruses are in a constant state of evolution due to antigenic drift occurring in the HA surface glycoprotein, which allows them to evade immunity present in the human population. It is important to characterize the genetic and antigenic changes that result from this drift to ensure the selection of optimal vaccine strains and to evaluate the effectiveness of vaccines against emerging variants [7,8].

Vaccination is the most effective way to prevent influenza-related illnesses, complications, and deaths, with an estimated 39% to 75% reduction in total mortality. Accordingly, the WHO recommends 75% vaccination coverage for the elderly, a vulnerable risk category [1,9]. Despite these, seasonal influenza nevertheless results in 3–5 million severe cases and 290,000–650,000 deaths worldwide annually, with 28,000–73,000 of those deaths taking place in Europe alone [10].

The surface glycoproteins hemagglutinin (HA) and neuraminidase (NA) serve as crucial targets for the host immune system, allowing it to differentiate between subtypes of the IAV [11]. HA surface proteins assist in the attachment of the virus to sialic acid receptors, which facilitates the entry of the virus via endocytosis and subsequent fusion with the host cell membrane. The NA surface protein, which acts as an enzyme that disrupts receptors, is important for both viral release and the intercellular propagation of viruses [12]. The HA protein of the influenza virus is cleaved into HA1 and HA2 components by the host cell protease enzyme. Modifications in the amino acid residues of HA1 can lead to changes in the binding preference of the influenza virus and may provide resistance against strain-specific antibodies that aim at the globular head of the HA protein [13]. Furthermore, it may lead to a discrepancy between the existing vaccine and the prevalent strain of influenza. Therefore, annual monitoring of the influenza virus is essential for collecting critical data required for the annual reformulation of influenza vaccines and for identifying any potential epidemic or pandemic [14].

Saudi Arabia is home to a large foreign workforce that frequently crosses its borders. Alongside the pilgrims visiting Makkah and Madinah year-round, particularly during the Hajj season, Saudi Arabia is regarded as a key area for the emergence and transmission of respiratory viruses. Thus, there is an immediate necessity for persistent surveillance of the influenza A/H3N2 subtype in Saudi Arabia. A few studies have outlined the epidemiology of the influenza A/H3N2 subtype in the previous year’s [15]. For instance, a recent study has demonstrated that IAV is the primary type of influenza virus in the Western region of Saudi Arabia over four epidemic seasons (2015–2019), noting that there were more cases of influenza A/H3N2 (42.0%) than A/H1N1 (27.3%) during the 2015–2018 seasons [16]. An additional study revealed that influenza A A/H3N2 was reported in a higher percentage of patients presenting with flu-like symptoms, showing 11.7% for A/H3N2 and 5.5% for the A/H1N1 subtype in Riyadh from 2019 to 2020 [17].

We have reported earlier the identification of the influenza A/H3N2 subtype in 43 (48.8%) from a total of 88 positive samples, while the other 45 (51.2%) were determined to be of the A/H1N1 subtype during five epidemic years (2014–2020) in Riyadh, Saudi Arabia [18]. Although these studies have shed light on the epidemiology of the influenza A/H3N2 subtype in Saudi Arabia, the knowledge regarding the molecular epidemiology and genetic diversity of this virus in the nation is still significantly uncharted. Continuous genetic and antigenic monitoring is crucial for tracking mutations that influence antigenicity, especially in regions with limited data. Thus, the current research intends to investigate the molecular epidemiology and circulation patterns of the influenza A/H3N2 subtype in Riyadh, Saudi Arabia, throughout the winter period of 2020–2023. This research seeks to clarify the molecular variations and phylogenetic connections of the locally circulating influenza A/H3N2 subtype in Riyadh, Saudi Arabia, during the winter season of 2020–2023 by conducting a genetic analysis of the essential *HA* and *NA* genes. Identifying amino acid substitutions, especially in key antigenic sites, and monitoring the circulation of genetic clades and subclades offer vital insights for the selection of vaccine strains and readiness for upcoming epidemics. Global surveillance of the influenza virus is crucial for detecting variants, including those that escape vaccines and those associated with antigenic shifts. Therefore, this study seeks to evaluate the molecular characterization and phylogenetic analysis of the *HA* and *NA* genes of the influenza A/H3N2 virus.

## 2. Materials and Methods

### 2.1. Study Population, Consent Forms, and Ethics

This study was conducted on 380 nasopharyngeal aspirates (NPAs) from patients admitted to King Khalid University Hospital (KKUH) in Riyadh, Saudi Arabia, Saudi Arabia, during the winter period (November to the following March) of the years 2020/21, 2021/22, and 2022/23. Samples were collected from patients residing in Riyadh at the time of admission, primarily Saudis and resident expatriates, excluding transient visitors such as pilgrims. The inclusion criteria specified that patients must present with fever (>38 °C) and respiratory symptoms, including cough, sore throat, and runny nose. The randomization was carried out using simple random sampling methods. Essential epidemiological information gathered included the ages of the patients, their stopover sites, and the dates of sample collection. The study protocol and sample collection procedures were approved by the Research Ethics Committee (RES) of King Saud University in Riyadh (Ethics Reference No. 22/0957/IRB).

### 2.2. Identification and Typing of IAV

RNA extraction was achieved using QIAamp viral RNA kit (Qiagen, Hilden, Germany, Cat. No. 52906), following the manufacturer’s instructions. IAV was identified through the OneStep Ahead reverse transcription polymerase chain reaction (RT-PCR) Kit utilizing Taq High Fidelity DNA Polymerase (Qiagen, Hilden, Germany, Cat. No. 220213). The RT-PCR reactions were performed using a Gene-Amp 9700 thermal cycler (Applied Biosystems, Foster City, CA, USA). The primer sets and RT-PCR methodology are described in our previous study [18].

### 2.3. Amplification of HA and NA Genes

The samples that reacted positively in the typing reactions were subjected to another round of RT-PCR for sequencing of the full-length *HA* and *NA* genes in IAV-positive samples using the same kit and primer sets as described in our recent study [18]. The analysis of PCR products was conducted using electrophoresis. Fourteen samples were chosen for sequencing via the Sanger method. All amplified fragments were sequenced on both strands at Macrogen Inc. (Macrogen, Seoul, Republic of Korea).

### 2.4. DNA Sequencing of HA and NA Genes

A total of 129 strains of the *HA* and *NA* genes from influenza A/H3N2 were gathered from the Global Initiative of Sharing All Influenza Data (GISAID) database and the National Center for Biotechnology Information (NCBI): (i) full-length, high-quality *HA* and *NA* gene sequences; (ii) a wide range of geographic regions and collection years to monitor spatiotemporal patterns; (iii) representative examples from all major viral clades and genotypes to guarantee a thorough evolutionary context; (iv) reference strains from the WHO vaccine for the Southern Hemisphere; (v) certain countries with significant numbers of pilgrims, visitors, and workers; and (vi) prototypes as reference strains. The strain A/New York/392/2004 isolated in the USA during 2004 was set as the consensus sequence. The Bioedit tool, version 7.2 (Ibis Biosciences, Carlsbad, CA, USA), was used to process full-length HA and NA nucleotide sequences via editing, assembly, alignment mutation, site identification, and amino acid change prediction.

Deduced amino acid sequences (Riyadh and international strains) of both HA and NA proteins were used to determine potential sites for O- and N-glycosylation sites via Net-N-glyc 1.0 https://services.healthtech.dtu.dk/services/NetOGlyc-4.0/ and Net-O-glyc 3.1 and https://services.healthtech.dtu.dk/services/NetNGlyc-1.0/ (accessed on 11 October 2025), respectively [19,20]. Conserved and new potential sites for N- and O-glycosylation were recorded.

Both the *HA* and *NA* genes were used to build phylogenetic trees using the Neighbor-Joining (NJ) method of Molecular Evolutionary Genetics Analysis (MEGA) X (Pennsylvania State University, University Park, PA, USA). Phylogenetic analysis was carried out and evaluated by 1000 bootstrap resampling iterations. The MEGA X software (version 10.0) was used to generate *p*-distance values to compute the intra-genotypic and inter-genotypic distances. Sequences of *HA* and *NA* genes in our study have been deposited into NCBI with the accession numbers (*HA* gene: PX446342-PX446355 and *NA* gene: PX446357-PX446370).

### 2.5. Statistical Analysis

The statistical analysis was performed using the Statistical Package for the Social Sciences (SPSS, version 22.0) statistical program (SPSS Inc., Chicago, IL, USA) version 22.0. The independent samples *t*-test was used to compare data that were regularly distributed across two groups. Frequencies were compared using the χ2 test. Statistical significance was defined as a *p*-value of less than 0.05.

## 3. Results

### 3.1. Demographic Analysis and IAV Prevalence

The prevalence of IAV was found to be 65 (17.11%) of 380 clinical samples, with 170 (44.74%) males and 210 (55.26%) females. that were included in the study during the epidemic season (winter 2020–21, 2021/22, and 2022–23). Additionally, A/H3N2 strains were more prevalent, 35 (9.21%), than A/H1N1 pdm09, 30 (7.89%). Regarding the results depending on gender, males were more affected, 44 (25.88%), compared to females, 21 (10.00%), (*p* < 0.05). Age categories were divided based on who is most likely to have influenza-related issues [21]. Ages 0–4 years, 5–14 years, 15–64 years, and 65 years and older were the four composite groups that were created. Significantly, 27 (28.42) of the total sample size consisted of 0–4-year-olds with a significant prevalence of IAV (*p* < 0.05). Data on A/H1N1 pdm09 and A/H3N2 strains incidence in different categories are shown in Table 1.

### 3.2. Analysis of Nucleotide and Amino Acid Sequences of the HA Gene

Nucleotide and amino acid sequence analysis was performed on the A/H3N2 strains using the consensus sequence (A/New York/392/2004). This analysis involved comparing the complete *HA* gene sequence (1701 nucleotides) circulating in Riyadh during the epidemic seasons of 2020–2023 with 129 homologous sequences from local and international strains sourced from various countries, including reference strains for vaccines established by the WHO (Table S1). Compared to the reference strains of A/New York/392/2004, the *HA* gene of A/H3N2 strains in this study revealed 11 significant substitutions, while the *NA* gene of the A/H1N1 pdm09 virus displayed 81-point mutations, accounting for 4.76%. These mutations were identified in the *HA* gene of Riyadh strains. Of these mutations, 21 changed their corresponding amino acids. Certain amino acid changes were consistently observed among the strains under examination, as indicated in Table 2.

### 3.3. Sequence Homology of IAV A/H3N2 HA and NA Genes

The sequences of A/H3N2 *HA* and *NA* genes were compared with the current vaccine strain (influenza A/Croatia/10136RV/2023). The sequence analysis indicated that the nucleotide identity varied from 97.8 to 98.1% (HA), whereas the NA identity was observed to be between 97.9 and 98.8%.

### 3.4. Comparative Analysis of RBD’s Antigenicity-Related Homologous Sequences

**Figure 1:**
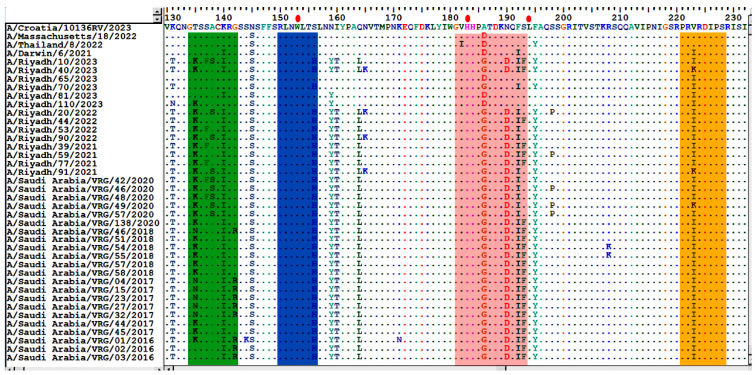
Alignment of the deduced amino acid sequences of the *HA1* gene. The alignment was performed using the Bioedit program, version 7.2. Different residues are represented by a single-letter code, while identical residues are indicated by dots. The enclosed green rectangle represents the 130-loop (residues 134–138); the enclosed blue rectangle represents the 150-loop (residues 150–156); the enclosed pink rectangle represents the 190-helix (residues 181–193); and the enclosed orange rectangle represents the 220-loop (residues 221–228). Red dots indicate conserved residues.

Comparative sequence analysis of the *HA* gene’s RBD was performed between the Riyadh strains and vaccine strains (A/Croatia/10136RV/2023, A/Massachusetts/18/2022, A/Thailand/8/2022, and A/Darwin/6/2021). This comparison revealed several homologous sequences within the RBD that are critical for antigenicity, including the 130-loop (residues 134–138), the 150-loop (residues 150–156), the 190-helix (residues 181–193), and the 220-loop (residues 221–228) (Figure 1).

**Figure 2:**
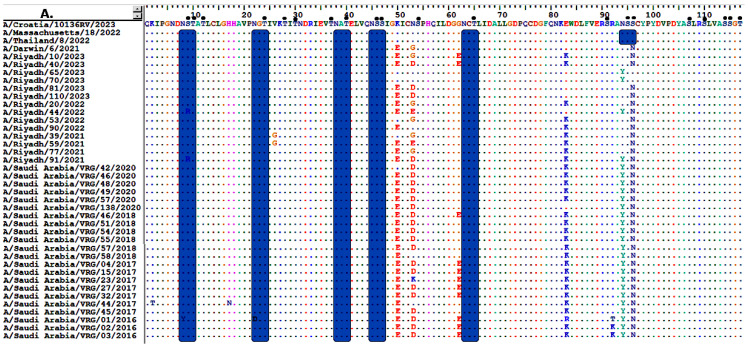

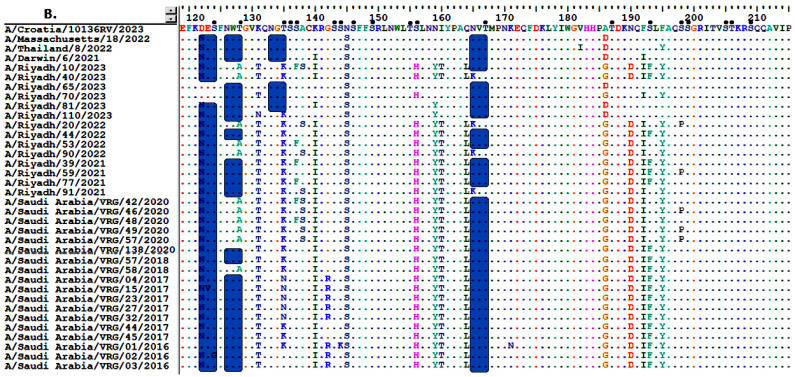

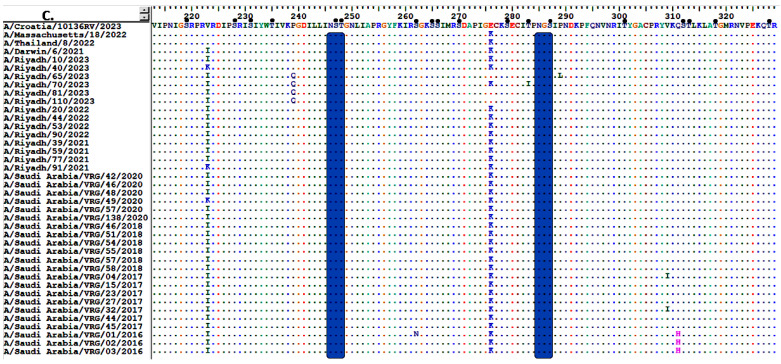
Alignment of the deduced amino acid sequences of the HA gene of Riyadh A/H3N2 strains and vaccine strain (A/Croatia/10136RV/2023, A/Thailand/8/2022, and A/Darwin/6/2021). (**A**) amino acid residues from 1 to 117, (**B**) from 119 to 215, and (**C**) from 213 to 330. Dots indicate identical residues, and different residues are shown in a single-letter code. Predicted N-linked glycosylation sites are enclosed in pink rectangles. Small, filled black circles correspond to predicted O-linked glycosylation sites.

The HA1 domain, comprising 1005 nucleotides, of Riyadh A/H3N2 strains was also compared with the current vaccine strains of A/H3N2, which include A/Croatia/10136RV/2023, A/Thailand/8/2022, and A/Darwin/6/2021. The results indicated that there were 20 point mutations (K50E, N53G/D, E83K, N94Y, ST96N, D122N, T128A, T135K, K140I, N145S, S156H, N159Y, I160T, Q164L, A168D/G, N190D, F192I, S193F, V223I/K, and E276K) that were permanently modified in the majority of strains from Riyadh, among which 9 were absent in all vaccine strains (E83K, T128A, T135K, S156H, N159Y, I160T, Q164L, N190D, and F192I), as illustrated in Figure 2A–C.

### 3.5. Sequence Analysis of the NA Gene

Regarding the full-length of the *NA* gene sequences (1410 nucleotides) of Riyadh A/H3N2 strains. A total of 42–55-point mutation (2.97–3.90%) were identified; 27 of these were reported to change their corresponding amino acids, and 23 were changed permanently (Table 3).

**Figure 3:**
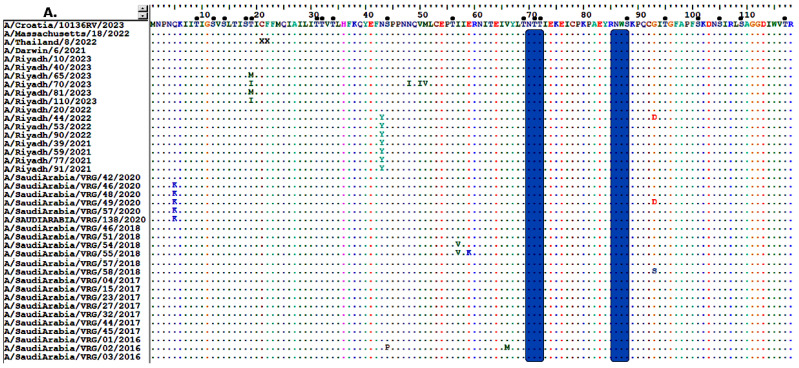

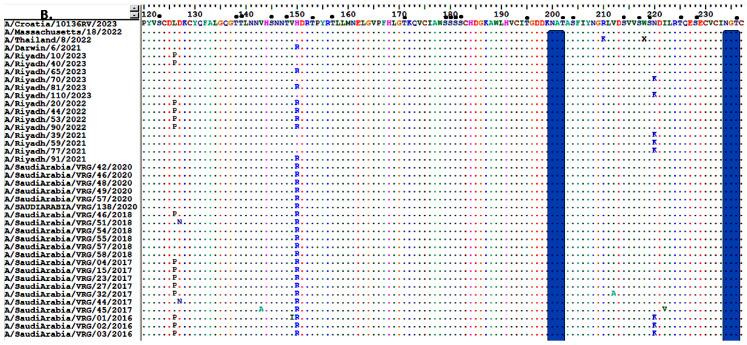

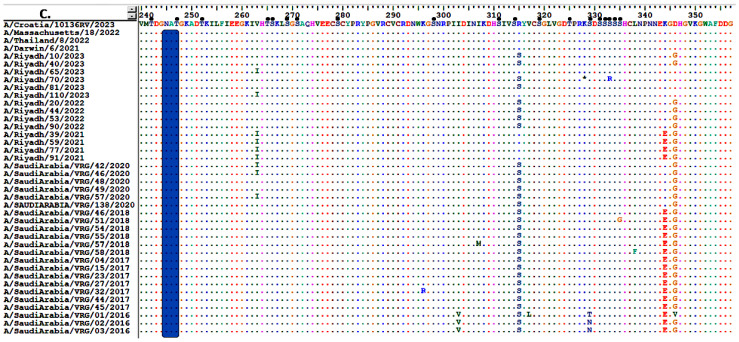

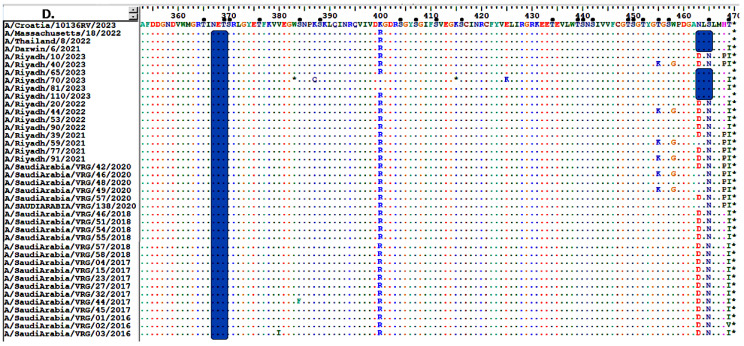
Alignment of the deduced amino acid sequences of the NA gene of Riyadh A/H3N2 strains and vaccine strain (A/Croatia/10136RV/2023, A/Thailand/8/2022, and A/Darwin/6/2021). (**A**) amino acid residues from 1 to 118, (**B**) from 120 to 237, (**C**) from 240 to 357, and (**D**) from 358 to 470. Dots indicate identical residues, and different residues are shown in a single-letter code. Predicted N-linked glycosylation sites are enclosed in pink rectangles. Small, filled black circles correspond to predicted O-linked glycosylation sites.

Comparison of the NA amino acid sequences of Riyadh A/H3N2 with the current vaccine strains (A/Croatia/10136RV/2023, A/Thailand/8/2022, and A/Darwin/6/2021) revealed 24-point mutations (1.70%). Nine of these amino acid residues (L126P, H137R, R315S, D346G, K400R, N463D, S465N, H468P, and T469I) had been permanently changed in the majority of Riyadh strains (Figure 3A–D).

### 3.6. Glycosylation Profiles of HA and NA Proteins

The HA protein of Riyadh strains has a considerable number of O-glycosylation sites, between 50 and 56. In contrast, a few numbers of N-glycosylation sites (*n* = 8 sites) were observed (8 NSTA, 22 NGTI, 34 NATE, 45 NSSI, 63 NCTL, 122 NESF, 246 NSTG, and 285 NGSI). Each of these potential N-glycosylation sites was also reported in vaccine strains (A/Croatia/10136RV/2023, A/Massachusetts/18/2022, A/Thailand/8/2022, and A/Darwin/6/2021). The vaccine strains A/Croatia/10136RV/2023, A/Thailand/8/2022, and A/Darwin/6/2021 were identified to have an additional N-glycosylation site (94 NSSC) that was absent from all strains from Riyadh. The vaccine strain exhibits N-glycosylation (94 NSSC), whereas our strains have mutated and substituted Y for N, resulting in the deletion of this N-glycosylation from our A/H3N2 strains (YSNC), which is not regarded as a site for N-glycosylation. Furthermore, three additional potential N-glycosylation sites (126 NWTG, 133 NGTS, 165 NVTM) have been reported in the vaccine strains (A/Croatia/10136RV/2023, A/Thailand/8/2022, and A/Darwin/6/2021), but these have not been observed in all strains analyzed in this research, as depicted in Figure 2A–C.

Regarding the NA protein of Riyadh, A/H3N2 strains, it is predicted that there are 6 N-glycosylation sites (70 NTTI, 86 NWSK, 200 NATA, 234 NGTC, 245 NATG, and 367 NETS), which are also found in the vaccine strains (A/Croatia/10136RV/2023, A/Thailand/8/2022, and A/Darwin/6/2021). Additionally, an extra N-glycosylation site (460 NLSL) was identified in the vaccine strains, which was exclusively found in A/Riyadh/65/2023, A/Riyadh/70/2023, and A/Riyadh/81/2023. In contrast, the O-glycosylation sites varied between 73 and 77 sites, as represented in (Figure 3A–D).

### 3.7. Phylogenetic Analysis

**Figure 4:**
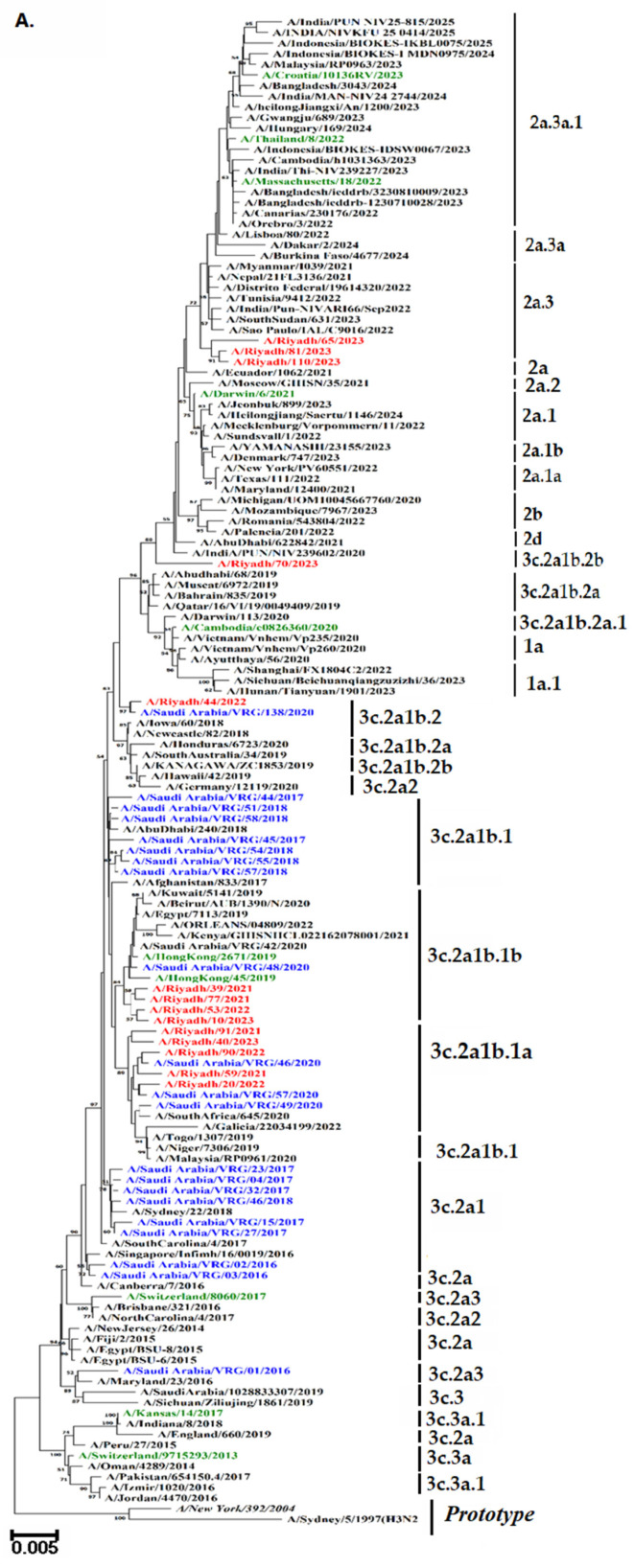

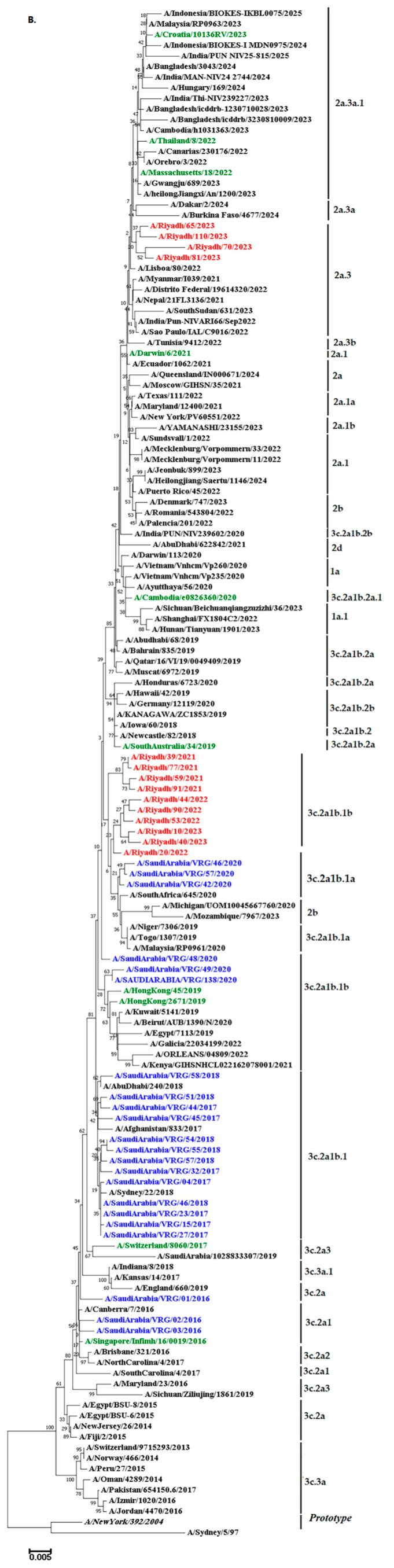
Phylograms of A/H3N2 strains: (**A**) *HA* gene (**B**) *NA* gene. The Neighbor-Joining method of the MEGA X program was used to build the phylogenetic tree based on nucleotide sequences. The numbers at the internal nodes of the tree represent the bootstrap values of 1000 replicates. Only values exceeding 50% are shown. Riyadh isolates identified in the current study are shown in blue. Those in purple represent historical Riyadh isolates. Those in green represent the WHO vaccine reference strains for the southern hemisphere. Reference and prototype strains are denoted by italic font.

To analyze the genetic diversity and relationships among our strains, phylogenetic trees of the *HA* and *NA* genes were plotted, along with a comparison to reference and vaccine strains, as shown in Figure 4A,B. It is noteworthy that the majority of Riyadh A/H3N2 strains were categorized within sub-clade 3c.2a1b.1a (*n* = 5). This sub-clade displayed the highest genetic similarity of 89% to several of our archived strains. Interestingly, some of Riyadh A/H3N2 strains (*n* = 4) were classified within sub-clade 3c.2a1b.1b, demonstrating sequence homology with the A/Hong Kong/45/2019 vaccine strain as well as some of our archived strains. Additionally, three more strains were categorized with sub-clade 2a.3 (*n* = 3) and 3c.2a1b.2b (*n* = 1), along with 3c.2a1b.2 (*n* = 1).

## 4. Discussion

The annual incidence of influenza requires continuous monitoring to detect changes that may cause human pandemics early on and to provide guidance to health officials on how to incorporate virus strains into seasonal vaccines properly. The current study aims to identify influenza virus subtypes and to evaluate their epidemiological features, genetic variation, and evolutionary dynamics in Riyadh, Saudi Arabia, across three winter seasons from 2020 to 2023, to inform policy decisions on influenza vaccines.

Our previous study found that the overall prevalence of IAV was 28.3% (88 out of 311), A/H1N1 pdm09 strains were more prevalent than A/H3N2 strains in Riyadh, Saudi Arabia, accounting for up to 45 (51.2%) of the 88 IAV-positive samples during the five winter seasons (2014–18 and 2019–20) [18]. In contrast to these findings, our current study revealed that over three winter seasons in Riyadh from 2020 to 2023, the total prevalence of IAV was 65 (17.11%) out of 380 samples, with the A/H3N2 strain considerably more prevalent 35 (9.21%) than the A/H1N1 pdm09 30 (7.89%). Our findings of A/H3N2 strain dominance were supported by a prior study carried out in the Western region of Saudi Arabia, which revealed that throughout the four seasons from October 2015 to 2019, influenza A/H3N2 strains were more common in 42%of cases, followed by A/H1N1 pdm09 strains in 27.3% of cases [16]. Likewise, the rate of IAV was found to be 151 (88%) out of 778 outpatients suffering from influenza-like illness. Among these cases, 91 (11.7%) were classified as A (H3N2) and 43 (5.5%) as A/H1N1 pdm09 at King Fahad Medical City (KFMC), in Riyadh, Saudi Arabia, during the 2019/2020 [17]. This trend agrees with the results from Al-Dorzi et al., indicating that in the epidemic year of 2021–2022, IAV was responsible for 533 of the 675 influenza cases (79%). A/H3N2 cases increased to 244 (36.1%), while H1N1 cases were noted at 161 (23.9%) for A/H1N1 [22]. The observed variations in circulation patterns may be ascribed to the significant mutation rate connected to the A/H3N2 strain and its relationship with local epidemic incidents [23].

Investigating the distribution of positive samples with respect to gender and age revealed some inconclusive predictions. IAV was more frequently observed in males 44 (25.88%) than in females 21 (10.00%), (*p* < 0.05). The rate of incidence for the A/H3N2 subtype is significantly higher in the age group (0–4 years), which is 14 (14.75%), compared to the age groups of 5–14, 15–64, and ≥65 years (*p* < 0.05). These findings contrast with our previous investigation, which showed that A/H3N2 strains were more common in females than in males (28.8% vs. 27.7%). The prevalence of A/H3N2 is substantially greater in the 15–64 age group than in the 0–4, 5–14, and ≥65 age groups (*p* < 0.05) [18]. In western Saudi Arabia, the A/H3N2 subtype was more prevalent in females (51.0%) among adult patients aged 19–60 throughout the four seasons from October 2015 to 2019 [16]. Another study conducted on a sample of rural Egyptians from 2017 to 2020 found that A/H3N2 strains were more common in females (55.0%) in the same age range [24].

IAV uses the HA peplomers on its viral envelope to initiate the infection process. The HA receptor-binding site binds the virus to surface glycoconjugates with terminal sialic acid residues when it reaches a possible host cell. The HA1 subunit, which has receptor-binding sites, is in charge of attaching to sialic acid receptors and causing endocytosis to induce viral entry [25]. The structure of the underlying oligosaccharide, the accessibility and abundance of receptors on the cell surface, the HA affinity for terminal sialic acid residues, and the protein or lipid moieties of the receptors all influence how well the virus binds to the host cell surface. Changes in influenza binding preference and antigenic transformation can result from modifications to amino acid residues in the receptor-binding domain of HA1 [26]. On the other hand, resistance and virulence are mostly linked to amino acid changes in the NA protein [27]. The changed viral antigenicity may be explained by a point mutation at nucleotide 126 in the HA1 domain sequence and the replacement of the matching amino acid residue [28].

RBD, which serves as the principal domain for binding to cellular receptors, is found at the membrane-distal end (HA1) of each HA monomer, spanning less than 300 amino acids (from around residue 105 to approximately residue 319, depending on the specific HA). The RBD site is made up of several critical structural components of HA1 for H3, such as the 130-loop (residue 134–142), the 150-loop (residue 150–156), the 190-helix (residue 181–193), and the 220-loop (residue 220–230), in addition to several conserved residues at the W153, H183, and L194 positions. By interacting with the sialic acid receptors, these characteristics, which differ among viral strains, affect the virus’s capacity to attach to and infect host cells. These highly variable regions are important targets for immune system-produced neutralizing antibodies in response to vaccination and disease [29].

Vaccination remains the most effective way to prevent and reduce influenza-related morbidity and mortality. The WHO recommends 75% vaccine coverage for older adults, who are more vulnerable [30]. The effectiveness of the current influenza vaccine is suboptimal, being estimated as 40% to 60% when the vaccine’s strains are antigenically well-matched with the circulating viruses [31,32]. The WHO organizes and leads vaccine strain consultation workshops twice a year for IAV vaccines to choose human immunization strains for the Northern or Southern Hemispheres [33]. Over 28 vaccine strains are available from the WHO for both the northern and southern hemispheres in an effort to improve the vaccination’s effectiveness and protection [18]. To evaluate the effectiveness of the existing vaccine against prevalent (A/H3N2) strains in Riyadh, we compared our strains with the vaccine strains A/Croatia/10136RV/2023, A/Massachusetts/18/2022, A/Thailand/8/2022, and A/Darwin/6/2021. Notably, our A/H3N2 strains exhibited 9 amino acid substitutions (E83K, T128A, T135K, S156H, N159Y, I160T, Q164L, N190D, and F192I) in the HA1 domain that were absent in all vaccine strains. These findings align with earlier studies that reported the nine mutations—E83K, T128A, T135K, S156H, N159Y, I160T, Q164L, N190D, and F192I in A/H3N2 strains contributed to a suboptimal match with vaccine strains [34,35]. The 2014–2015 H3N2 viruses have HA changes at L3I, N144S, N145S, F159Y, K160T, N225D, and Q311H, T128A, A138S, R142G, N145S, F159S, and N225D when compared to the A/Texas/50/2012 strain [34].

In our previous study, the root 3c.2a contained A/Saudi Arabia/VRG-02/2016, A/Saudi Arabia/VRG-03/2016, A/New Jersey/26/2014, A/Fiji/2/2015, and A/Canberra/7/2016. Thirteen more strains belonging to subclade 3C.2a1b were identified by mutations of F159Y, K160T, N171K, N121K, K92R, and H311Q [34,36]. The 3c. clades/subclade comprised 16 strains in total; two members of Clade 3c.2a (A/Saudi Arabia/VRG-2/2016 and A/Saudi Arabia/VRG-7/2016) were identified by the mutations F159Y and K160T [34,37]. With the exception of A/Saudi Arabia/VRG-01/2016, which belonged to clade 3c.2a3 and had no vaccination stains, the strains under investigation remained in the same clade as the vaccine strains.

The glycosylation of the HA and NA proteins of influenza plays a vital role in evading the host’s immune responses [38]. For instance, the addition of N-linked glycosylation from IAV to antibody binding sites on seasonal A/H3N2 may inhibit antibody attachment to HA and NA by concealing antigenic determinants [39]. N-glycosylation sites in the Riyadh strains HA proteins were eight (8 NSTA, 22 NGTI, 34 NATE, 45 NSSI, 63 NCTL, 122 NESF, 246 NSTG, and 285 NGSI). Additionally, the vaccination strains A/Croatia/10136RV/2023, A/Massachusetts/18/2022, A/Thailand/8/2022, and A/Darwin/6/2021 all had these N/glycosylation sites. Additionally, three other putative N-glycosylation sites (126 NWTG, 133 NGTS, and 165 NVTM) have been identified in the vaccination strains (A/Croatia/10136RV/2023, A/Thailand/8/2022, and A/Darwin/6/2021), albeit not all of the strains examined in this study had these. Whether or how this change may have reduced the virus’s virulence in response to the vaccine is unknown. These results are consistent with a previous study conducted in Yokohama, Japan in 2016–17 and 2017–18, which found that certain viruses in the clade 3C.2A1b had the T135K or T135N substitution, which resulted in the shift in the glycosylation site at positions 135–137 (NSS) but the loss of the glycosylation site at positions 133–135 (NGK or NGN) [40]. Sites 144-NNSF and 483-NGTY had high proportions of complex N-glycans in Phil82, and the similarities to their corresponding sites in Phil82gal were <0.01 and 0.03, respectively [41].

Furthermore, what are the consequences of the previously indicated adaptation of seasonal A/H3N2 viruses to humans if this mutation results in it? Will selection pressure cause a new pandemic to break out, or will the vaccination fail to stop the newly circulating strains? Further study is needed to answer these problems.

A phylogenetic tree was constructed to observe the possible circulation pattern of the influenza A/H3N2 subtype over time, using *HA* and *NA* genes of vaccine strains and globally circulating strains. It is noteworthy that the majority of Riyadh A/H3N2 strains were categorized within sub-clade 3c.2a1b.1a (*n* = 5). Followed by sub-clade 3c.2a1b.1b, (*n* = 4). These clades displayed the highest genetic similarity of 89% to several of our archived strains in Riyadh during the epidemic years of 2014–2020 [18]. The analysis’s findings showed that our A/Riyadh A/H3N2 strains did not exclusively cluster with the vaccine strains that the WHO recommended for use in 2021–2023: A/Croatia/10136RV/2023, A/Massachusetts/18/2022, A/Thailand/8/2022, and A/Darwin/6/2021. These variations may be explained by the fact that many viral introductions appear to have a major impact on evolution within local epidemics, allowing different clades of the same subtype to circulate simultaneously [42,43].

This study’s primary limitation is the small cross-sectional sample size. Furthermore, it is unable to identify the reason for the recurrence of infection. Further rigorous investigations with higher sample sizes throughout various regions of Saudi Arabia during repeated epidemic seasons are necessary to have a more comprehensive picture of the circulation trends of A/H3N2.

## 5. Conclusions

In conclusion, our study documented the genetic variation, vaccination strain match, and prevalence in Riyadh. The results showed that 9.21% of all samples contained A/H3N2 strains in circulation, which was much more common. The majority of the positive samples were males (16.47%), and the most afflicted group was the 0–4 age group (14, 14.75%). Nine of the twenty amino acid substitutions found in the HA1 domain of A/H3N2 strains were not present in any of the vaccine strains. The predominant strain of A/H3N2 obtained from Riyadh was classified within the sub-clade 3c.2a1b.1a and 3c.2a1b.1b, which exhibited no exclusive clustering with the strains utilized in the vaccine. The Riyadh HA protein has 8–11 N-glycosylation sites, some of which have been observed in vaccine strains but are absent from all strains evaluated in this study. The examination of the sequence and phylogenetic characteristics of A/H3N2 viral strains has indicated the existence of characteristic features, specific amino acid substitutions, and changes in N-glycosylation sites, in comparison to the vaccine strains currently utilized for managing influenza epidemics in the Northern Hemisphere. As a result, the flu vaccines administered in Saudi Arabia might need to be reevaluated to incorporate additional vaccine strains that are more pertinent to those currently circulating in the recent epidemic seasons in Saudi Arabia.

## Figures and Tables

**Table 1:** Sample distribution across epidemic seasons (winters 2020/21, 2021/22, and 2022/23), gender, and age groups.

		No. of samples N (%)	Positive for IAV N (%)	Positive for	
				A/H1N1 pdm09 N (%)	A/H3N2 N (%)
Total		380	65 (17.11)	30 (7.89)	35 (9.21)
Season	2020/21	120 (31.57)	14 (11.67)	6 (5.00)	8 (6.67)
	2021/22	130 (34.21)	17(14.17)	6 (4.62)	11 (8.46)
	2022/23	130 (34.21)	34 (26.15)	18 (13.84)	16 (12.30)
Gender	Male	170 (44.74)	44 (25.88) ^a^	16 (9.41) ^a^	28 (16.47) ^a^
	Female	210 (55.26)	21 (10.00)	14 (6.67)	7 (3.33)
Age in years	0–4	95 (20.00)	27 (28.42) ^b^	13 (13.68) ^b^	14 (14.75) ^b^
	5–14	110 (28.95)	19 (17.27)	7 (6.35)	12 (10.91)
	15–64	120 (31.57)	9 (7.50)	4 (3.33)	5 (4.16)
	≥65	55 (14.48)	10 (18.18)	6 (10.91)	4(7.27)

Data are displayed as percentages (%). ^a^ Significantly higher (*p* < 0.05) than males. ^b^ Significantly higher (*p* < 0.05) than age groups 5–14, 15–64, and ≥65 years.

**Table 2:** Mutations of the *HA* gene of the study A/H3N2 strains as compared with the reference strain (A/New York/392/2004).

Mutation Site	19	49	61	64	66	73	108	110	136	151	156	160	174	189	232	294	328	377	390	420	466	500	505
A/New York/392/2004	L	Q	S	T	G	E	K	Y	N	T	K	N	K	K	S	N	N	T	N	I	R	G	D
A/Riyadh/10/2023	I	R	N	I	E	.	R	N	K	K	I	S	N	Q	N	K	S	R	D	V	K	E	N
A/Riyadh/40/2023	I	R	N	I	E	.	R	N	K	K	I	S	N	Q	N	K	S	R	D	V	K	E	N
A/Riyadh/65/2023	I	R	N	I	K	G	R	.	K	.	.	S	N	Q	N	K	S	R	D	V	K	E	N
A/Riyadh/70/2023	I	R	N	I	K	G	R	.	K	.	.	S	N	Q	N	K	S	R	D	V	K	E	N
A/Riyadh/81/2023	I	R	N	I	E	G	R	N	K	.	I	S	N	Q	N	K	S	R	D	V	K	E	N
A/Riyadh/110/2023	I	R	N	I	E	G	R	N	K	K	.	S	N	Q	N	K	S	R	D	V	K	E	N
A/Riyadh/20/2022	I	R	N	I	E	G	R	N	K	K	I	S	N	Q	N	K	S	R	D	V	K	E	N
A/Riyadh/44/2022	I	R	N	I	E	G	R	.	K	K	I	S	N	Q	N	K	S	R	D	V	K	E	N
A/Riyadh/53/2022	I	R	N	I	K	G	R	N	K	K	I	S	N	Q	N	K	S	R	D	V	K	E	N
A/Riyadh/90/2022	I	R	N	I	E	G	R	N	K	K	I	S	N	Q	N	K	S	R	D	V	K	E	N
A/Riyadh/39/2021	I	R	N	I	K	G	R	N	K	K	I	S	N	Q	N	K	S	R	D	V	K	E	N
A/Riyadh/59/2021	I	R	N	I	E	G	R	N	K	K	I	S	N	Q	N	K	S	R	D	V	K	E	N
A/Riyadh/77/2021	I	R	N	I	E	G	R	N	K	K	I	S	N	Q	N	K	S	R	D	V	K	E	N
A/Riyadh/91/2021	I	R	N	I	E	G	R	.	K	K	I	S	N	Q	N	K	S	R	D	V	K	E	N
A/Saudi Arabia/VRG/42/2020	I	R	N	I	K	G	R	.	K	K	I	S	N	Q	N	K	S	R	D	V	K	E	N
A/Saudi Arabia/VRG/46/2020	I	R	N	I	E	G	R	.	K	K	I	S	N	Q	N	K	S	R	D	V	K	E	N
A/Saudi Arabia/VRG/48/2020	I	R	N	I	E	G	R	.	K	K	I	S	N	Q	N	K	S	R	D	V	K	E	N
A/Saudi Arabia/VRG/49/2020	I	R	N	I	E	G	R	.	K	K	I	S	N	Q	N	K	S	R	D	V	K	E	N
A/Saudi Arabia/VRG/57/2020	I	R	N	I	E	G	R	.	K	K	I	S	N	Q	N	K	S	R	D	V	K	E	N
A/Saudi Arabia/VRG/138/2020	I	R	N	I	E	G	R	.	K	K	I	S	N	Q	N	K	S	R	D	V	K	E	N
A/Saudi Arabia/VRG/46/2018	I	R	N	I	E	E	R	.	K	N	I	S	N	Q	N	K	S	R	D	V	K	E	N
A/Saudi Arabia/VRG/51/2018	I	R	N	I	E	G	R	.	K	K	I	S	N	Q	N	K	S	R	D	V	K	E	N
A/Saudi Arabia/VRG/54/2018	I	R	N	I	E	G	R	.	K	K	I	S	N	Q	N	K	S	R	D	V	K	E	N
A/Saudi Arabia/VRG/55/2018	I	R	N	I	E	G	R	.	K	K	I	S	N	Q	N	K	S	R	D	V	K	E	N
A/Saudi Arabia/VRG/57/2018	I	R	N	I	E	G	R	.	K	K	I	S	N	Q	N	K	S	R	D	V	K	E	N
A/Saudi Arabia/VRG/58/2018	I	R	N	I	E	G	R	.	K	K	I	S	N	Q	N	K	S	R	D	V	K	E	N
A/Saudi Arabia/VRG/04/2017	I	R	N	I	E	.	R	.	K	N	I	S	N	Q	N	K	S	R	D	V	K	E	N
A/Saudi Arabia/VRG/15/2017	I	R	N	I	E	.	R	.	K	N	I	S	N	Q	N	K	S	R	D	V	K	E	N
A/Saudi Arabia/VRG/23/2017	I	R	N	I	E	.	R	.	K	N	I	S	N	Q	N	K	S	R	D	V	K	E	N
A/Saudi Arabia/VRG/27/2017	I	R	N	I	E	.	R	.	K	N	I	S	N	Q	N	K	S	R	D	V	K	E	N
A/Saudi Arabia/VRG/32/2017	I	R	N	I	E	.	R	.	K	N	I	S	N	Q	N	K	S	R	D	V	K	E	N
A/Saudi Arabia/VRG/44/2017	I	R	N	I	E	G	R	.	K	K	I	S	N	Q	N	K	S	R	D	V	K	E	N
A/Saudi Arabia/VRG/45/2017	I	R	N	I	E	G	R	.	K	K	I	S	N	Q	N	K	S	R	D	V	K	E	N
A/Saudi Arabia/VRG/01/2016	I	R	N	I	E	.	T	.	K	K	I	K	N	Q	N	K	S	R	D	.	K	.	N
A/Saudi Arabia/VRG/02/2016	I	R	N	I	E	.	.	.	K	.	I	S	N	Q	N	K	S	R	D	V	K	E	N
A/Saudi Arabia/VRG/03/2016	I	R	N	I	E	.	.	.	K	.	I	S	N	Q	N	K	S	R	D	V	K	E	N

Dots indicate identical residues and different residues are shown in a single-letter code. Predicted N-linked glycosylation sites are enclosed in pink rectangles. Small, filled black circles correspond to predicted O-linked glycosylation sites.

**Table 3:** Mutations of the *NA* gene of the study A/H3N2 strains as compared with the reference strain (A/New York/392/2004).

Mutation Site	43	77	81	93	126	147	150	194	215	220	221	245	247	263	267	303	310	329	339	344	367	464	505
A/New York/392/2004	N	M	L	D	P	D	H	V	I	K	K	S	S	I	T	V	Y	N	D	E	S	I	P
A/Riyadh/10/2023	.	I	P	G	.	N	.	I	V	N	D	N	T	V	K	I	H	S	N	K	N	L	.
A/Riyadh/40/2023	.	I	P	G	.	N	.	I	V	N	D	N	T	V	K	I	H	S	N	K	N	L	.
A/Riyadh/65/2023	.	I	P	G	L	N	R	I	V	N	D	N	T	I	K	I	H	S	N	K	N	L	H
A/Riyadh/70/2023	.	I	P	G	L	N	.	I	V	.	D	N	T	V	K	I	H	S	N	K	N	L	H
A/Riyadh/81/2023	.	I	P	G	L	N	R	I	V	N	D	N	T	V	K	I	H	S	N	K	N	L	H
A/Riyadh/110/2023	.	I	P	G	L	N	.	I	V	K	D	N	T	.	K	I	H	S	N	K	N	L	H
A/Riyadh/20/2022	.	I	P	G	.	N	R	I	V	N	D	N	T	V	K	I	H	S	N	K	N	L	H
A/Riyadh/44/2022	Y	I	P	.	.	N	R	I	V	N	D	N	T	V	K	I	H	S	N	K	N	L	H
A/Riyadh/53/2022	Y	I	P	G	.	N	R	I	V	N	D	N	T	V	K	I	H	S	N	K	N	L	H
A/Riyadh/90/2022	Y	I	P	G	.	N	R	I	V	N	D	N	T	V	K	I	H	S	N	K	N	L	H
A/Riyadh/39/2021	Y	I	P	G	L	N	.	I	V	.	D	N	T	.	K	I	H	S	N	.	N	L	.
A/Riyadh/59/2021	Y	I	P	G	L	N	.	I	V	.	D	N	T	.	K	I	H	S	N	.	N	L	.
A/Riyadh/77/2021	Y	I	P	G	L	N	.	I	V	.	D	N	T	.	K	I	H	S	N	.	N	L	.
A/Riyadh/91/2021	Y	I	P	G	L	N	R	I	V	N	D	N	T	.	K	I	H	S	N	.	N	L	.
A/Saudi Arabia/VRG/42/2020	.	I	P	G	L	N	R	I	V	N	D	N	T	.	K	I	H	S	N	K	N	L	.
A/Saudi Arabia/VRG/46/2020	.	I	P	G	L	N	R	I	V	N	D	N	T	.	K	I	H	S	N	K	N	L	.
A/Saudi Arabia/VRG/48/2020	.	I	P	G	L	N	R	I	V	N	D	N	T	V	K	I	H	S	N	K	N	L	.
A/Saudi Arabia/VRG/49/2020	.	I	P	.	L	N	R	I	V	N	D	N	T	V	K	I	H	S	N	K	N	L	.
A/Saudi Arabia/VRG/57/2020	.	I	P	G	L	N	R	I	V	N	D	N	T	.	K	I	H	S	N	K	N	L	.
A/Saudi Arabia/VRG/138/2020	.	I	P	G	L	N	R	I	V	N	D	N	T	V	K	I	H	S	N	K	N	L	.
A/Saudi Arabia/VRG/46/2018	.	I	P	G	.	N	R	I	V	N	D	N	T	V	K	I	H	S	N	.	N	L	H
A/Saudi Arabia/VRG/51/2018	.	I	P	G	L	N	R	I	V	N	D	N	T	V	K	I	H	S	N	.	N	L	H
A/Saudi Arabia/VRG/54/2018	.	I	P	G	L	N	R	I	V	N	D	N	T	V	K	I	H	S	N	.	N	L	H
A/Saudi Arabia/VRG/55/2018	.	I	P	G	L	N	R	I	V	N	D	N	T	V	K	I	H	S	N	.	N	L	H
A/Saudi Arabia/VRG/57/2018	.	I	P	G	L	N	R	I	V	N	D	N	T	V	K	I	H	S	N	.	N	L	H
A/Saudi Arabia/VRG/58/2018	.	I	P	S	L	N	R	I	V	N	D	N	T	V	K	I	H	S	N	.	N	L	H
A/Saudi Arabia/VRG/04/2017	.	I	P	G	.	N	R	I	V	N	D	N	T	V	K	I	H	S	N	.	N	L	H
A/Saudi Arabia/VRG/15/2017	.	I	P	G	.	N	R	I	V	N	D	N	T	V	K	I	H	S	N	.	N	L	H
A/Saudi Arabia/VRG/23/2017	.	I	P	G	.	N	R	I	V	N	D	N	T	V	K	I	H	S	N	.	N	L	H
A/Saudi Arabia/VRG/27/2017	.	I	P	G	.	N	R	I	V	N	D	N	T	V	K	I	H	S	N	.	N	L	H
A/Saudi Arabia/VRG/32/2017	.	I	P	G	.	N	R	I	V	N	D	N	T	V	K	I	H	S	N	.	N	L	H
A/Saudi Arabia/VRG/44/2017	.	I	P	G	L	N	R	I	V	N	D	N	T	V	K	I	H	S	N	.	N	L	H
A/Saudi Arabia/VRG/45/2017	.	I	P	G	L	N	R	I	V	N	D	N	T	V	K	I	H	S	N	.	N	L	H
A/Saudi Arabia/VRG/01/2016	.	I	P	G	.	N	R	I	V	.	D	N	T	V	K	.	H	T	N	.	N	L	H
A/Saudi Arabia/VRG/02/2016	.	I	P	G	.	N	R	I	V	.	D	N	T	V	K	.	H	.	N	.	N	L	H
A/Saudi Arabia/VRG/03/2016	.	I	P	G	.	N	R	I	V	.	D	N	T	V	K	.	H	.	N	.	N	L	H

The same residues of amino acids are shown by dots. Amino acids presented in red show the unique mutations of the *NA* gene of the study strains.

## Data Availability

The datasets generated and analyzed during the current study are available from the corresponding author upon reasonable request. The sequence data reported in this study have been deposited in GenBank under accession numbers (*HA* gene: PX446342-PX446355 and *NA* gene: PX446357-PX446370).

## Supplementary Materials

The following supporting information can be downloaded at: https://www.mdpi.com/article/10.3390/vaccines13121184/s1. Table S1: List of A/H3N2 strains included in sequence and phylogenetic analysis.
