# Supplementary material for: Evolution and Vaccine Strain Match of HA and NA Genes of Influenza A/H3N2 Subtype in Riyadh, Saudi Arabia, 2020–2023
Source: Vaccines (Basel). 2025 Nov 22;13(12):1184. doi: 10.3390/vaccines13121184 (PMC12737434; doi:10.3390/vaccines13121184)
Supplement: Supplementary file 1 [file vaccines-13-01184-s001.zip › vaccines-3965489-supplementary.pdf]

Table S1: List of A/H3N2 strains included in sequence and phylogenetic analysis

No.	Strain	Origin	Collection Date	Gisaid Accession No. HA	NA	Gisaid Clade
1.	A/New York/392/2004	USA/Ref.	2004	EPI252225	EPI79013	3c.2a
2.	A/NewJersey/26/2014	USA	2014	EPI841441	EPI841440	3c.2a
3.	A/Fiji/2/2015	New Zealand	2015	EPI636655	EPI636655	3c.2a
4.	A/Canberra/7/2016	Australia	2016	EPI866694	EPI793167	3c.2a1
5.	A/Singapore/Infimh/16/0019/2016	Singapore	2015	EPI1381186	EPI1381185	3c.2a1
6.	A/SouthCarolina/4/2017	USA	2017	EPI1094351	EPI1094350	3c.2a1
7.	A/Sydney/22/2018	Australia	2018	EPI1251865	EPI1251864	3c.2a1b.1
8.	A/Afghanistan/833/2017	Afghanistan	2017	EPI1197229	EPI1197228	3c.2a1b.1
9.	A/AbuDhabi/240/2018	UAE	2018	EPI1245538	EPI1245537	3c.2a1b.1
10.	A/SouthAfrica/645/2020	South Africa	2020	EPI1759254	EPI1759253	3c.2a1b.1a
11.	A/Niger/7306/2019	Niger	2019	EPI1651919	EPI1651918	3c.2a1b.1a
12.	A/Togo/1307/2019	Togo	2019	EPI1683617	EPI1683616	3c.2a1b.1a
13.	A/Malaysia/RP0961/2020	MalaysiA	2020	EPI1759278	EPI1759277	3c.2a1b.1a
14.	A/HongKong/45/2019	China	2019	EPI1691930	EPI1691929	3c.2a1b.1b
15.	A/HongKong/2671/2019	China	2019	EPI1592035	EPI1592034	3c.2a1b.1b
16.	A/Egypt/7113/2019	Egypt	2019	EPI1882614	EPI1882615	3c.2a1b.1b
17.	A/Kuwait/5141/2019	Kuwait	2019	EPI1719377	EPI1719376	3c.2a1b.1b
18.	A/Beirut/AUB/1390/N/2020	Lebanon	2020	EPI1814744	EPI1814743	A
19.	A/Iowa/60/2018	USA	2018	EPI1324969	EPI1359998	3c.2a1b.2
20.	A/Newcastle/82/2018	UK	2018	EPI1595444	EPI1595443	3c.2a1b.2
21.	A/SouthAustralia/34/2019	Australia	2019	EPI1607117	EPI1607116	3c.2a1b.2a
22.	A/Qatar/16/VI/19/0049409/2019	Qatar	2019	EPI1619516	EPI1619517	3c.2a1b.2a
23.	A/Abudhabi/68/2019	UAE	2019	EPI1636325	EPI1636324	3c.2a1b.2a
24.	A/Muscat/6972/2019	Oman	2019	EPI1646754	EPI1646900	3c.2a1b.2a
25.	A/Bahrain/835/2019	Bahrain	2019	EPI1696676	EPI1696675	3c.2a1b.2a
26.	A/Cambodia/e0826360/2020	CambodiA	2020	EPI1843589	EPI1843588	3c.2a1b.2a.1
27.	A/Darwin/113/2020	Australia	2020	EPI1733843	EPI1733842	3c.2a1b.2a.1
28.	A/Vietnam/Vnhcm/Vp260/2020	Vietnam	2020	EPI1848008	EPI1848007	1a
29.	A/Darwin/6/2021	Australia	2021	EPI1885402	EPI1885401	2a.1
30.	A/Michigan/UOM10045667760/2020	USA	2020	EPI2095215	EPI2095213	2b
31.	A/IndiA/PUN/NIV239602/2020	IndiA	2020	EPI1843873	EPI1843872	3c.2a1b.2b
32.	A/KANAGAWA/ZC1853/2019	Japan	2019	EPI1398386	EPI1398385	3c.2a1b.2b
33.	A/Hawaii/42/2019	USA	2019	EPI1486401	EPI1486400	3c.2a1b.2b
34.	A/Germany/12119/2020	Germany	2020	EPI1756862	EPI1756861	3c.2a2
35.	A/Brisbane/321/2016	Australia	2016	EPI919282	EPI919281	3c.2a2
36.	A/NorthCarolina/4/2017	USA	2017	EPI925625	EPI925624	3c.2a2
37.	A/Switzerland/8060/2017	Switzerland	2017	EPI1326015	EPI1326014	3c.2a3
38.	A/Maryland/23/2016	USA	2016	EPI868424	EPI868423	3c.2a3
39.	A/SaudiArabia/1028833307/2019	Saudi Arabia	2019	EPI1754145	EPI1754146	3c.2a3
40.	A/Sichuan/Ziliujing/1861/2019	China	2019	EPI1648634	EPI1648633	3c.3
41.	A/Oman/4289/2014	Oman	2014	EPI551900	EPI551901	3c.3a
42.	A/Izmir/1020/2016	Turkiye	2016	EPI829311	EPI829312	3c.3a
43.	A/Jordan/4470/2016	Jordan	2016	EPI769557	EPI769558	3c.3a
44.	A/Switzerland/9715293/2013	Switzerland	2013	EPI814528	EPI814527	3c.3a
45.	A/Egypt/BSU-8/2015	Egypt	2015	MG745925	MG745928	3c.3a
46.	A/Peru/27/2015	Peru	2015	EPI629582	EPI629581	3c.3a
47.	A/Egypt/BSU-6/2015	Egypt	2015	MG745923	MG745926	3c.3a
48.	A/Kansas/14/2017	USA	2017	EPI1504535	EPI1504534	3c.3a.1
49.	A/Indiana/8/2018	USA	2018	EPI1197165	EPI1197164	3c.3a.1
50.	A/England/660/2019	UK	2019	EPI1741059	EPI1741058	3c.2a

51.	A/ORLEANS/04809/2022	France	2022	EPI1999181	EPI1999180	3c.2a1b.1b
52.	A/Galicia/22034199/2022	Spain	2022	EPI2287489	EPI2287488	3c.2a1b.1a
	A/Kenya/GIHSNHCL022162078001/2021	Kenya	2021	EPI2251286	EPI2251285	3c.2a1b.1b
53.	A/YAMANASHI/23155/2023	Japan	2023	EPI3045768	EPI3045767	2a.1b
54.	A/New_York/PV60551/2022	USA	2022	EPI2433730	EPI2433728	2a.1a
55.	A/Jeonbuk/899/2023	South Korea	2023	EPI2716104	EPI2716103	2a.1
	A/Indonesia/BIOKES-I_MDN0975/2024	Indonesia	2024	EPI4052700	EPI4052699	2a.3a.1
56.	A/Dakar/2/2024	Senegal	2024	EPI3426277	EPI3426271	2a.3a.1
57.	A/Romania/543804/2022	Romania	2022	EPI2341258	EPI2341260	2b
	A/India/PUN_NIV25-815/2025	India	2025	EPI4529120	EPI4529119	2a.3a.1
58.	A/Mecklenburg/Vorpommern/33/2022	Germany	2022	EPI2010873	EPI2010872	2a.1
59.	A/Bangladesh/icddrb/3230810009/2023	Bangladesh	2023	EPI3501178	EPI3501176	2a.3a.1
	A/Indonesia/BIOKES-IKBL0075/2025	Indonesia	2025	EPI4609402	EPI4609401	2a.3a.1
60.	A/Tunisia/9412/2022	Tunisia	2022	EPI2177198	EPI2177197	2a.3b
61.	A/Shanghai/FX1804C2/2022	CHINA	2022	EPI3589876	EPI3589874	1a.1
62.	A/Myanmar/I039/2021	Myanmar	2021	EPI1998579	EPI1998578	2a.3
63.	A/Sydney/5/1997	Pakistan	1997	KM821316	AJ291403	2a.3
	A/India/Thi-NIV239227/2023	India	2023	EPI3930633	EPI3930632	2a.3a.1
64.	A/Canarias/230176/2022	Spain	2022	EPI2400126	EPI2400125	2a.3a.1
65.	A/Puerto_Rico/45/2022	Puerto Rico	2022	MZ819914.	EPI2347601	2a.1
66.	A/Honduras/6723/2020	Honduras	2020	EPI1801213	EPI1801212	3C.2a1b.2
67.	A/Sichuan/Beichuanqiangzuzizhi/36/2023	China	2023	EPI2669304	EPI2669303	1a.1
68.	A/Denmark/747/2023	Denmark	2023	EPI2530234	EPI2530233	2a.1b
69.	A/Burkina_Faso/4677/2024	Burkina Faso	2024	EPI3607338	EPI3607337	2a.3a
70.	A/Ayutthaya/56/2020	Thailand	2020	EPI1852221	EPI1852220	1a
71.	A/Mozambique/7967/2023	Mozambique	2023	EPI2677083	EPI2677081	2B
72.	A/Nepal/21FL3136/2021	Nepal	2021	EPI1998591	EPI1998590	2a.3
73.	A/Gwangju/689/2023	South Korea	2023	EPI2454901	EPI2454900	2a.3a.1
74.	A/Texas/111/2022	USA	2022	EPI3346872	EPI3346871	2a.1a
	A/INDIA/NIVKFU_25_0414/2025	India	2025	EPI4759437	EPI4759436	2a.3a.1
	A/India/Pun-NIVARI66/Sep2022	India	2022	EPI2414159	EPI2414158	2a.3
75.	A/SouthSudan/631/2023	South Sudan	2023	EPI2760859	EPI2762757	2a.3
76.	A/heilongjiangxi/An/1200/2023	China	2023	EPI2668810	EPI2668809	2a.3a.1
77.	A/Thailand/8/2022	Thailand	2022	EPI2236266	EPI2236265	2a.3a.1
78.	A/Indonesia/BIOKES-IDSW0067/2023	Indonesia	2023	EPI3009496	EPI3009495	2a.3a.1
79.	A/Bangladesh/3043/2024	Bangladesh	2024	EPI3295041	EPI3295040	2a.3a.1
	A/India/MAN-NIV24_2744/2024	India	2024	EPI4246876	EPI4246875	2a.3a.1
80.	A/AbuDhabi/622842/2021	UAE	2021	EPI1881257	EPI1885008	2d
81.	A/Massachusetts/18/2022	USA	2022	EPI2413620	EPI2413618	2a.3a.1
82.	A/Ecuador/1062/2021	Ecuador	2021	EPI2003445	EPI2003444	2a
83.	A/Cambodia/h1031363/2023	Cambodia	2023	EPI2976861	EPI2976858	2a.3a.1
84.	A/Palencia/201/2022	Spain	2022	EPI2932394	EPI2932393	2 b
85.	A/Distrito_Federal/19614320/2022	Brazil	2022	EPI3042949	EPI3042948	2a.3
86.	A/Bangladesh/icddrb-1230710028/2023	Bangladesh	2023	EPI3500617	EPI3500616	2a.3a.1
87.	A/Malaysia/RP0963/2023	Malaysia	2023	EPI2976995	EPI2976994	**2a.3a.1**
88.	A/Moscow/GIHSN/35/2021	Russia	2021	EPI1965272	EPI1965274	2a.2
89.	A/Pakistan/654150.6/2017	Pakistan	2017	MZ819914	EPI2010832	2a.1
90.	A/Orebro/3/2022	Sweden	2022	EPI2220434	EPI2220433	2a.3a.1
91.	A/Gan/Tianyuan/1901/2023	China	2023	EPI2822284	EPI2822283	1a.1
92.	A/Lisboa/80/2022	Portugal	2022	EPI2021273	EPI2021272	2a.3a
93.	A/Vietnam/Vnhcm/Vp235/2020	Vietnam	2020	EPI1847904	EPI1847903	1a
94.	A/Maryland/12400/2021	USA	2021	EPI1925992	EPI1925991	2a.1a
95.	A/Sundsvall/1/2022	Sweden	2022	EPI2020806	EPI2020805	2a.1
96.	A/Heilongjiang/Saertu/1146/2024	China	2024	EPI3466808	EPI3466807	2a.1
97.	A/Sao_Paulo/IAL/C9016/2022	Brazil	2022	EPI1997075	EPI1997074	2a.3

98.	A/Croatia/10136RV/2023	Croatia	2023	EPI3472940	EPI3472932	**2a.3a.1**
99.	A/Hungary/169/2024	Hungary	2024	EPI3447507	EPI3447509	2a.3a.1
100.	A/Saudi Arabia/VRG/01/2016	Saudi Arabia	2016	ON514565	ON514227	
101.	A/Saudi Arabia/VRG/02/2016	Saudi Arabia	2016	ON514617	ON514616	
102.	A/Saudi Arabia/VRG/03/2016	Saudi Arabia	2016	ON520897	ON520901	
103.	A/Saudi Arabia/VRG/04/2017	Saudi Arabia	2017	ON521141	ON521167	
104.	A/Saudi Arabia/VRG/15/2017	Saudi Arabia	2017	ON521169	ON521191	
105.	A/Saudi Arabia/VRG/23/2017	Saudi Arabia	2017	ON521222	ON521223	
106.	A/Saudi Arabia/VRG/27/2017	Saudi Arabia	2017	ON522003	ON524169	
107.	A/Saudi Arabia/VRG/32/2017	Saudi Arabia	2017	ON524170	ON524408	
108.	A/Saudi Arabia/VRG/44/2017	Saudi Arabia	2017	ON627717	ON524821	
109.	A/Saudi Arabia/VRG/45/2017	Saudi Arabia	2017	ON524823	ON524824	
110.	A/Saudi Arabia/VRG/46/2018	Saudi Arabia	2018	ON524839	ON524860	
111.	A/Saudi Arabia/VRG/51/2018	Saudi Arabia	2018	ON524871	ON525112	
112.	A/Saudi Arabia/VRG/54/2018	Saudi Arabia	2018	ON527514	ON527515	
113.	A/Saudi Arabia/VRG/55/2018	Saudi Arabia	2018	ON527517	ON527519	
114.	A/Saudi Arabia/VRG/57/2018	Saudi Arabia	2018	ON527525	ON527526	
115.	A/Saudi Arabia/VRG/58/2018	Saudi Arabia	2018	ON527530	ON527548	
116.	A/Saudi Arabia/VRG/42/2020	Saudi Arabia	2020	OP536171	OP536586	
117.	A/Saudi Arabia/VRG/46/2020	Saudi Arabia	2020	OP536200	OP536588	
118.	A/Saudi Arabia/VRG/48/2020	Saudi Arabia	2020	OP536201	OP536589	
119.	A/Saudi Arabia/VRG/49/2020	Saudi Arabia	2020	OP536205	OP536988	
120.	A/Saudi Arabia/VRG/57/2020	Saudi Arabia	2020	OP536209	OP537019	
121.	A/Saudi Arabia/VRG/138/2020	Saudi Arabia	2020	OP536417	OP537028	
